# Different Anatomical Subsites of Colon Cancer and Mortality: A Population-Based Study

**DOI:** 10.1155/2018/7153685

**Published:** 2018-08-30

**Authors:** Xing-kang He, Wenrui Wu, Yu-e Ding, Yue Li, Lei-min Sun, Jianmin Si

**Affiliations:** ^1^Department of Gastroenterology, Sir Run Run Shaw Hospital, Zhejiang University School of Medicine, Hangzhou 310016, China; ^2^Institute of Gastroenterology, Zhejiang University (ZJU), Hangzhou 310016, China; ^3^State Key Laboratory for Diagnosis and Treatment of Infectious Diseases, The First Affiliated Hospital, School of Medicine, Zhejiang University, Hangzhou, China; ^4^Collaborative Innovation Centre for Diagnosis and Treatment of Infectious Diseases, Hangzhou, China

## Abstract

**Background:**

In terms of incidence and pathogenesis, right-sided colon cancer (RCC) and left-sided colon cancer (LCC) exhibit several differences. However, whether existing differences could reflect the different survival outcomes remains unclear. Therefore, we aimed to ascertain the role of location in the prognosis.

**Methods:**

We identified colon cancer cases from the Surveillance, Epidemiology, and End Results database between 1973 and 2012. Differences among subsites of colon cancer regarding clinical features and metastatic patterns were compared. The Kaplan-Meier curves were conducted to compare overall and disease-specific survival in relation to cancer location. The effect of tumour location on overall and cancer-specific survival was analysed by Cox proportional hazards model.

**Results:**

A total of 377,849 patients from SEER database were included in the current study, with 180,889 (47.9%) RCC and 196,960 (52.1%) LCC. LCC was more likely to metastasize to the liver and lung. Kaplan-Meier curves demonstrated that LCC patients had better overall and cancer-specific survival outcomes. Among Cox multivariate analyses, LCC was associated with a slightly reduced risk of overall survival (HR, 0.92; 95% CI, 0.92-0.93) and cancer-specific survival (HR, 0.92; 95% CI, 0.91-0.93), even after adjusted for other variables. However, the relationship between location and prognosis was varied by subgroups defined by age, year at diagnosis, stage, and therapies.

**Conclusions:**

We demonstrated that LCC was associated with better prognosis, especially for patients with distant metastasis. Future trails should seek to identify the underlying mechanism.

## 1. Introduction

Colorectal cancer remains the third common malignancy in males and the second in females worldwide, respectively [1]. In 2012, it was estimated that approximately 1,400,000 individuals were diagnosed with colorectal cancer, with accounting for 694,000 deaths globally [1]. Notably, screening tests and comprehensive treatments of colorectal cancer had contributed to the better prognosis in the past decades [2].

It is well acknowledged that colon and rectal cancer share several similarities; however, some important differences exist as well [3, 4]. Recently, colon cancer subsites, in terms of right- or left-sided origins, had aroused great public interests [5–11]. Several studies had investigated the influence of different anatomic sites in clinical features and survival outcomes of colon cancer [6, 8, 9, 12, 13]. Back in 1990, Bufill.et al. [14] firstly proposed that tumour located in the distal and proximal colon location might possess different biologic and genetic properties. Subsequently, accumulating evidence had indicated that right- and left-sided colon cancer (RCC and LCC) not only located on different sites simply but also presented distinct clinical symptoms and molecular profiles [10, 15–19]. According to previous studies, patients with RCC always presented with subtle or occult symptoms, higher tumour stage, poor differentiated, higher percentage of CIMP, MSI, and BRAF mutation positive [18, 20–23]. As opposed to RCC, patients with LCC frequently exhibited evident symptoms, lower tumour stages, and chromosomal instability [23–26]. The underlying mechanisms for these discrepancies were still uncertain, yet, different embryologic origins might partly account for those [12]. However, whether those differences could translate into different survival outcomes needs further exploration. Several studies had attempted to assess the prognostic role of cancer location in patients with colon cancer [5, 7, 11–13, 23]. However, the current results remained conflicting and might need to be further addressed. [13, 17, 27]. For example, in 2011, Weiss and colleagues [17] observed no overall survival difference between RCC and LCC among patients with combined I–III stages. In 2014, another study also reported that no survival advantage was observed among patients with either right- or left-sided stage II cancer [27]. The inconsistent association might reflect the complexity of this issue, limited sample size, and high degree of heterogeneity across studies.

In light of those conflicting results, we, therefore, performed a retrospective analysis to investigate the impact of tumour location on survival outcomes in a population-based study. Furthermore, we also examined whether the prognostic role of cancer location was influenced by different groups of age, stage, year of diagnosis, and therapies.

## 2. Results

### 2.1. Patient Characteristics

**Figure 1:**
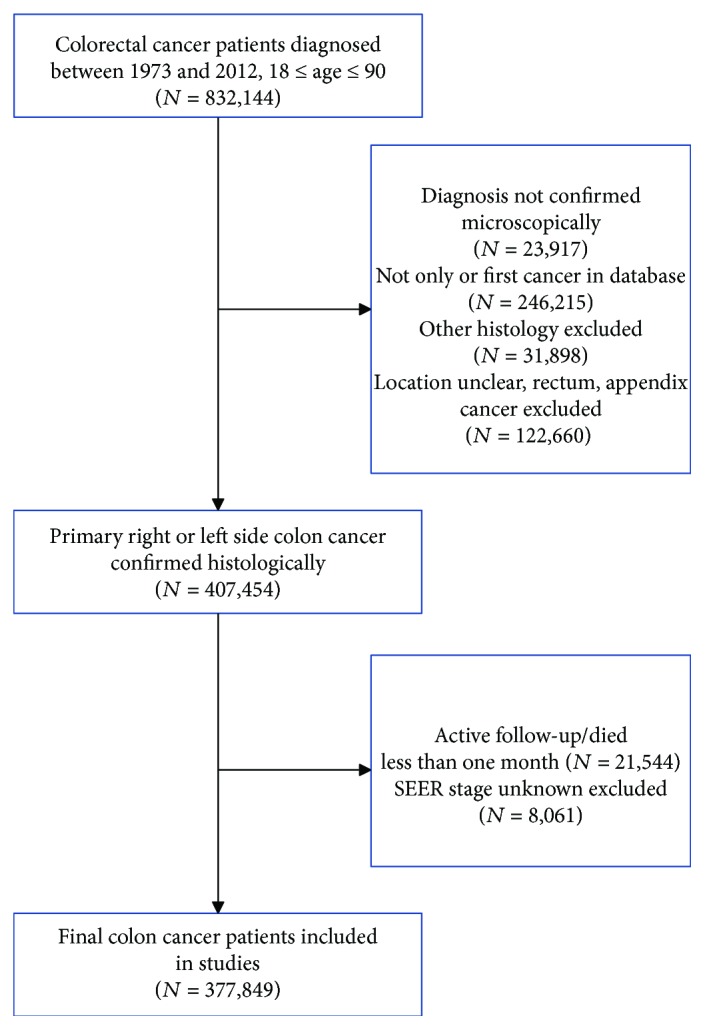
Flowchart for include patients from the Surveillance, Epidemiology, and End Results database.

A total of 377,849 cases with colon cancer during 1973 to 2012 were included in our studies. The detailed selection diagram of the population was presented in Figure 1. Among populations, there were 180,889 (47.9%) patients with RCC and 196,960 (52.1%) patients with LCC. The proportion of patients with RCC increased faster from 1973–1982 to 2003–2012 compared with LCC. Patients with RCC were older, female, and poorly differentiated.  Table 1 summarized the baseline characteristics with respect to primary cancer location.

### 2.2. Metastasis Pattern of Colorectal Cancer by Subsites

**Figure 2:**
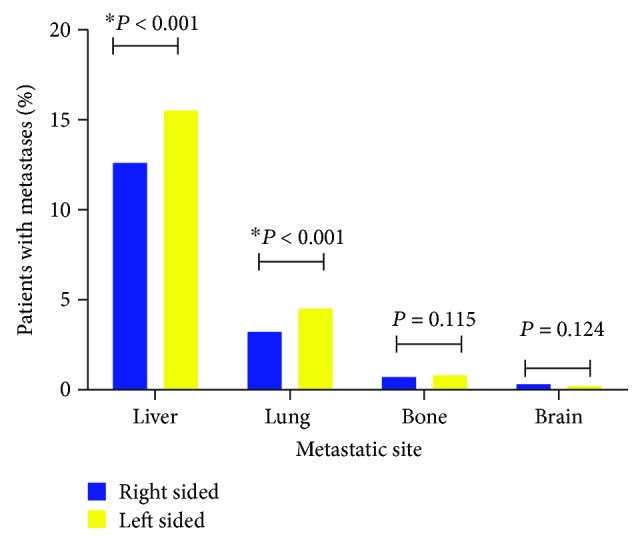
Metastatic distribution patterns between right- and left-sided colon cancers. ^∗^*P* < 0.01.

**Figure 3:**
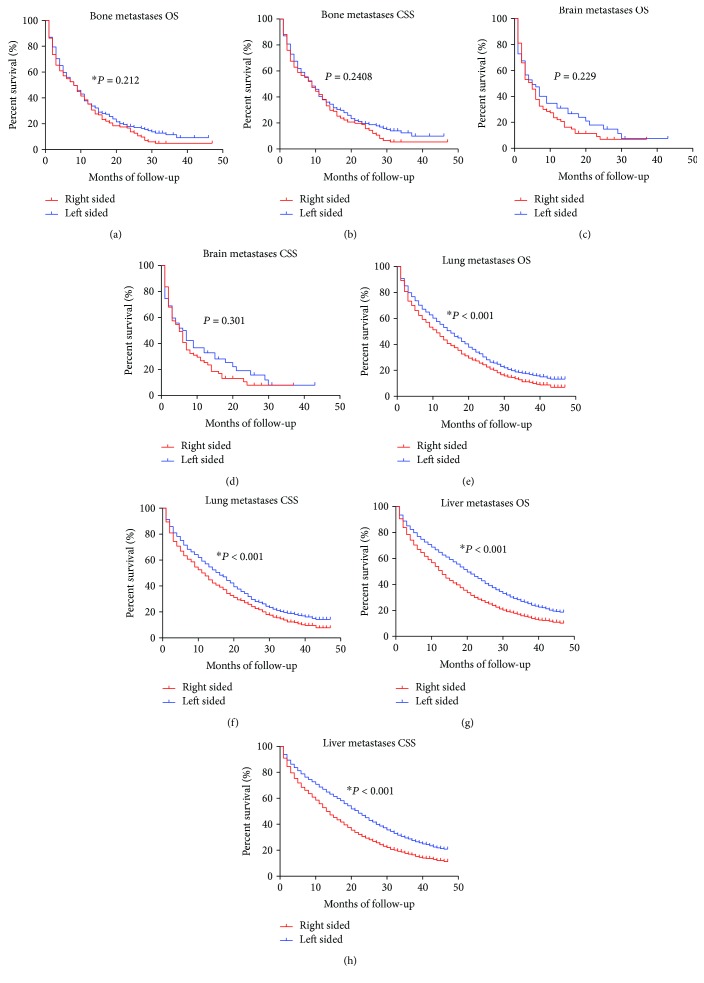
Kaplan-Meier survival analysis of patients with different metastases. (a) Overall survival for bone metastases. (b) Cancer-specific survival for bone metastases. (c) Overall survival for brain metastases. (d) Cancer-specific survival for brain metastases. (e) Overall survival for lung metastases. (f) Cancer-specific survival for lung metastases. (g) Overall survival for liver metastases. (h) Cancer-specific survival for liver metastases.

The SEER database only included detailed main information of four metastatic sites about the bone, brain, liver, and lung since 2010^+^. Therefore, we included patients with clear metastatic information from 2010 to 2012 and made use of those population to analyse the synchronous metastasis distribution according to primary cancer location. The most frequent distant metastasis site of colon cancer was the liver, followed by the lung, bone, and brain, in respective of subsites. As shown in Figure 2, patients with LCC were more likely to have liver and lung metastasis at the time of diagnosis (both *P* < 0.01), which were main distant metastases for colon cancer. However, there were no significant difference between RCC and LCC for bone and brain metastases (both *P* > 0.05). Furthermore, we analysed the OS and CSS between RCC and LCC stratified by different metastases. As shown in Figure 3, there was no OS and CSS difference among RCC and LCC for bone and brain metastases. For liver and lung metastasis, patients with LCC enjoyed a better OS and CSS in relation to those with RCC.

### 2.3. Survival Analysis of Cancer Subsites by Stage, Age, Year of Diagnosis, and Therapies

**Figure 4:**
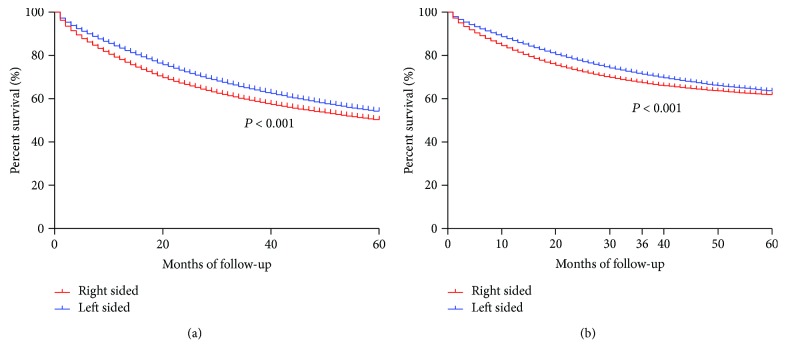
Overall and cancer-specific analysis between right- and left-sided colon cancers. (a) Overall survival. (b) Cancer-specific survival.

Kaplan-Meier curves firstly demonstrated a significant difference of OS and CSS between RCC and LCC patients (both *P* < 0.001, Figure 4). The overall five-year survival rates for RCC and LCC patients were 50.6% and 54.4%, respectively. Multivariable Cox analyses demonstrated that cancer location was an independent prognostic factor for OS and CSS, even after adjusting for other variables, such as age, year, gender, race, insurance, marital status, tumour stage, grade, surgery, radiation, and chemotherapy (RCC as reference, OS, LCC HR = 0.92, 95% CI, 0.92-0.93; CSS, adjusted HR = 0.92, 95% CI, 0.91-0.93) (Table 2). Furthermore, we analyses overall survival differences in subgroups defined by the age (i.e., <50 y, 50–69 y, ≥70 y), year at diagnosis (i.e., 1973–1982, 1983–1992, 1993–2002, and 2003–2012), SEER tumour stage (i.e., regional, localized, and distant), and therapies (surgery, radiation, and chemotherapy). Interestingly, we found that the prognostic effect of subsites was inconsistent across subgroups (Table 3). The benefits associated with cancer location were more pronounced in colon cancer diagnosed at 2003–2010. There were no significant survival advantages among localized and regional stages, while survival benefits associated with left-sided cancer were obvious among distant stage. By contrast, in older age group (≥70 y), patients with RCC even had a decreased risk of mortality in relation to LCC. For different therapies, the prognostic role of tumour location was consistent.

## 3. Discussion

Utilizing population-based database from SEER, we observed that different metastatic distribution and prognosis among right- and left-sided cancers. In accordance with previous studies [12], liver and lung metastases were more likely to present in left-sided carcinomas. It was reported that peritoneal metastases were in right-sided carcinomas predominately [12]. Since lacking other distant metastatic information, we were unable to examine this pattern. Subsequently, we demonstrated that LCC was significantly associated with better OS and CSS, even after adjusted for multiple variables. Our result was consistent with a large body of research, which indicated potential survival benefits existed among patients with left-sided cancer, although several studies could not confirm this result [17, 27]. Whether other factors influenced the prognostic effect of primary cancer location was less well studied. Therefore, we performed subgroup analysis to explore other factors. Notably, we observed the association was varied across different age, stage, year at diagnosis, and therapies. Patients with left-sided cancer were significantly associated with favourable overall survival, especially for patients with distant metastases. The clear underlying causes remained unknown. Different tumour biology and therapies might be partly accounted for those.

Recently, differences between RCC and LCC has aroused considerable attentions [3, 4, 11, 14, 23]. An increasing amount of evidence showed that RCC and LCC had differences in clinical presentation, pathology, and molecular signatures. According to Missiaglia and colleagues [28], microsatellite instable-high (MSI) and BRAF mutation were predominate among proximal (right-sided) tumours, while distal cancers (left-sided) were characterised by chromosome instable, high expression of epiregulin, and human epidermal growth factor receptor 2 (HER2) amplification [24, 29, 30]. Another large genome-scale analysis of colorectal cancer tissues conducted by the Cancer Genome Atlas Network also revealed some differences between cancers originating from the right colon and all other sites [31]. It is conceivable that underlying molecular base might drive the observed survival difference between RCC and LCC.

Taking colon cancer as a heterogeneous group with different genetic and epigenetic changes into account, appropriate classification of colorectal cancer is increasingly important for clinical practice, especially for therapies chosen in the exciting age of precision medicine [25]. Although molecular classification is promising, huge cost impedes its wide application. Whether primary cancer location could be considered as a surrogate marker for prognosis attracts huge interests, although the significance of cancer location on prognosis was still a dispute. Chemotherapy is an important part of advanced cancer treatments. It was reported that RCC and LCC exhibited different response to chemotherapy and targeted treatments, which might influence therapy selection. In 2013, a retrospective analysis of two independent cohorts indicated that only metastatic CRC patients with left-side cancer might benefit from bevacizumab in combination with capecitabine and oxaliplatin. [32] Further study by Loupakis et al. [6] validated this finding in three independent cohorts (PROVETTA, AVF2107g, and NO16966). However, in 2015, Brulé et al. [13] reanalysed the results of NCIC CO.17 trial and the results showed that tumour location was only predictive of progression-free survival benefit from cetuximab in refractory metastatic colon cancer, although location alone was absent of prognostic effect on survival in best supportive care group. In ASCO 2016, Venook and colleagues reanalysed CALGB/SWOG 80405 (Alliance) study and unexpectedly found that survival benefits associated with the side of colon appeared to be far greater in metastatic stage than previously considered [33]. A striking survival difference was observed in subgroups among RCC and LCC. Cetuximab was superior to bevacizumab for overall survival when the primary tumour was on the left side [33]. It indicated that the response of cetuximab and bevacizumab were dependent on the location of the primary tumours, which reminds us that anatomical location may promisingly indicate optimal therapy regimen selection. A recent meta-analysis by Petrelli et al. [34] including 66 studies demonstrated that left-sided colon cancer was associated with better prognosis (HR, 0.82; 95% CI, 0.79-0.84; *P* < 0.001) and colon cancer location should be considered as a prognostic criterion when making treatment decisions.

As a retrospective study, several intrinsic limitations of this study should be considered. Firstly, SEER dataset lacks of detailed information on specific chemotherapy regimens, especially for biotarget therapies. Therefore, we were unable to adjust this important effect on survival. Secondly, genetic or molecular marker statuses were not available in this dataset. We failed to examine the effect of molecular difference on survival of right- and left-sided cancers.

In summary, subsites of colon cancer could be potentially considered as an independent prognostic factor for OS and CSS. Additional further prospective research should verify this association and seek to elucidate the underlying biological mechanisms. We hope our finding could provide some evidence for further studies.

## 4. Methods

### 4.1. Data Sources and Cohort Definition

We identified patients with primary colon cancer from the Surveillance, Epidemiology, and End Results (SEER) between 1973 and 2012. Primary cancer site was identified by the International Classification of Diseases for Oncology (ICD-O-3) site codes (C18.0, C18.2 to C18.7, and C19.9), and adenocarcinoma type was identified by the ICD-O-3 histology codes (8140 to 8147, 8210 to 8211, 8220 to 8221, 8260 to 8263, 8480 to 8481, and 8490). In this analysis, we adopted the SEER historic staging system instead of the American Joint Committee on Cancer (AJCC) system because of its advantage that recorded consistently from 1973 to 2012. According to previous studies [6], we discriminated right and left-sided cancers by splenic flexure as the cut-off. Therefore, C18.0 (cecum), C18.2 (ascending colon), C18.3 (hepatic flexure of colon), and C18.4 (transverse colon) were considered as right-sided colon cancers, and C18.5 (splenic flexure of colon), C18.6 (descending colon), C18.7 (sigmoid colon), and 19.9 (recto-sigmoid) were defined as left-sided colon cancers. We defined any cause of deaths as events and alive as censored events in overall survival analysis. In cause-specific survival analysis, we defined deaths due to colon cancer as events and deaths from any other causes as censored events. In addition, we included patients who were diagnosed with colon cancer during 2010–2012 to analyse metastatic pattern. The following cases were excluded in our study: colon cancer was not the primary cancer; cases diagnosed at autopsy or by death certificate only and without histological confirmation; patients who died less than one month. This study was approved by the review board of the Sir Run Run Shaw Hospital, Zhejiang University School Medicine, Zhejiang, China.

### 4.2. Statistical Analysis

We conducted chi-square tests to compare the clinical characteristics and metastatic pattern between RCC and LCC. Kaplan-Meier curves were conducted to compare overall and cancer-specific survival between RCC and LCC within different metastasis sites. The multivariable Cox analyses were adopted to calculate corresponding hazard ratios (HRs) and 95% confidence intervals (CIs). According to previous studies, we selected several prognostic variables and confounders into Cox proportional hazards, such as age, year, gender, race, insurance, marital status, tumour stage, grade, surgery, radiation, and chemotherapy. Two-sided *P* values at the *P* < 0.05 level was considered to be statistically significant. All analyses were performed with SPSS version 20.0 (SPSS, Chicago, Illinois, USA).

## Figures and Tables

**Table 1:** Baseline patient and disease characteristics of patients by primary tumour location.

Characteristic	Right-sided tumours	Left-sided tumours	*P*
*Year at diagnosis*			*<0.001*
1973–1982	18,670 (10.3%)	26,094 (13.2%)	
1983–1992	25,442 (14.1%)	33,380 (16.9%)	
1993–2002	51,278 (28.3%)	54,272 (27.6%)	
2003–2012	85,499 (47.3%)	83,214 (42.2%)	
*Age at diagnosis* ^†^			*<0.001*
<50 y	13,333 (7.4%)	22,511 (11.4%)	
50–69 y	67,385 (37.3%)	93,511 (47.5%)	
≥70 y	100,171 (55.4%)	80,938 (41.1%)	
*Gender*			*<0.001*
Male	80,436 (44.5%)	103,612 (52.6%)	
Female	100,453 (55.5%)	93,348 (47.4%)	
*Race*			*<0.001*
White	149,172 (82.5%)	159,284 (80.9%)	
Black	20,626 (11.4%)	18,880 (9.6%)	
Other (AP, AI/AN)	10,545 (5.8%)	17,772 (9.0%)	
Unknown	546 (0.3%)	1024 (0.5%)	
*Insurance status*			*<0.001*
Insured	49,043 (27.1%)	45,911 (23.3%)	
Uninsured	1520 (0.8%)	1991 (1.0%)	
Unknown	130,326 (72.0%)	149,058 (75.7%)	
*Marital status*			*<0.001*
Married	97,678 (54.0%)	115,912 (58.9%)	
Unmarried	76,752 (42.4%)	73,159 (37.1%)	
Unknown	6459 (3.6%)	7889 (4.0%)	
*Tumour stage*			*<0.001*
Localized	65,978 (36.5%)	78,611 (39.9%)	
Regional	76,694 (42.4%)	76,269 (38.7%)	
Distant	38,217 (21.1%)	42,080 (21.4%)	
*Tumour grade* ^‡^			*<0.001*
I	16,562 (9.2%)	22,181 (11.3%)	
II	104,036 (57.5%)	120,896 (61.4%)	
III	38,575 (21.3%)	24,897 (12.6%)	
IV	2790 (1.5%)	1546 (0.8%)	
Unknown	18,926 (10.5%)	27,440 (13.9%)	
*Surgery*			*<0.001*
Surgery	173,582 (96.0%)	187,853 (95.4%)	
No surgery	7202 (4.0%)	8993 (4.6%)	
Unknown	105 (0.1%)	114 (0.1%)	
*Radiation*			*<0.001*
Radiation	3453 (1.9%)	15,658 (7.9%)	
No radiation	175,810 (97.2%)	179,168 (91.0%)	
Unknown	1626 (0.9%)	2134 (1.1%)	
*Chemotherapy*			*<0.001*
Yes	50,568 (28.0%)	59,160 (30.0)	
No	130,321 (72.0%)	137,800 (70.0%)	

^†^Year; ^‡^I means well differentiated; II means moderately differentiated; III means poorly differentiated; IV means undifferentiated. AP: Asian or Pacific Islander; AI/AN: American Indian/Alaska Native.

**Table 2:** Univariate and multivariate analysis of overall and cancer-specific survival in patients with colon cancer.

Variable	Overall survival				Cancer-specific survival			
	Univariate		Multivariate		Univariate		Multivariate	
	HR (95% CI)	*P*	HR (95% CI)	*P*	HR (95% CI)	*P*	HR (95% CI)	*P*
*Year of diagnosis*								
1973–1982	Reference		Reference		Reference		Reference	
1983–1992	0.88 (0.87, 0.89)	<0.001	0.87 (0.86, 0.89)	<0.001	0.82 (0.80, 0.83)	<0.001	0.82 (0.81, 0.84)	<0.001
1993–2002	0.74 (0.73, 0.75)	<0.001	0.73 (0.72, 0.74)	<0.001	0.67 (0.66, 0.68)	<0.001	0.64 (0.63, 0.66)	<0.001
2003–2012	0.59 (0.58, 0.59)	<0.001	0.62 (0.61, 0.63)	<0.001	0.54 (0.53, 0.55)	<0.001	0.52 (0.51, 0.53)	<0.001
*Age at diagnosis*								
<50 y	Reference		Reference		Reference		Reference	
50–69 y	1.34 (1.32, 1.37)	<0.001	1.48 (1.46, 1.51)	<0.001	1.02 (1.00, 1.04)	0.091	1.19 (1.17, 1.21)	<0.001
≥70 y	2.45 (2.41, 2.49)	<0.001	2.87 (2.82, 2.92)	<0.001	1.17 (1.15, 1.19)	<0.001	1.58 (1.55, 1.61)	<0.001
*Gender*								
Male	Reference		Reference		Reference		Reference	
Female	0.93 (0.93, 0.94)	<0.001	0.81 (0.80, 0.81)	<0.001	0.93 (0.92, 0.94)	<0.001	0.89 (0.88, 0.90)	<0.001
*Race*								
White	Reference		Reference		Reference		Reference	
Black	1.08 (1.07, 1.10)	<0.001	1.15 (1.13, 1.16)	<0.001	1.20 (1.18, 1.22)	<0.001	1.18 (1.17, 1.20)	<0.001
Other (AP, AI/AN)	0.77 (0.76, 0.78)	<0.001	0.86 (0.85, 0.88)	<0.001	0.84 (0.82, 0.85)	<0.001	0.91 (0.89, 0.93)	<0.001
*Primary site*								
Right	Reference		Reference		Reference		Reference	
Left	0.86 (0.85, 0.87)	<0.001	0.92 (0.92, 0.93)	<0.001	0.93 (0.92, 0.94)	<0.001	0.92 (0.91, 0.93)	<0.001
*Insurance status*								
Insured	Reference		Reference		Reference		Reference	
Uninsured	1.21 (1.14,1.28)	<0.001	1.21 (1.15,1.28)	<0.001	1.43 (1.35, 1.52)	<0.001	1.11 (1.04, 1.18)	<0.001
*Marital status*								
Unmarried	Reference		Reference		Reference		Reference	
Married	1.37 (1.36, 1.39)	<0.001	1.26 (1.25, 1.27)	<0.001	1.18 (1.17, 1.19)	<0.001	1.14 (1.12, 1.15)	<0.001
*Tumour stage*								
Localized	Reference		Reference		Reference		Reference	
Regional	1.59 (1.57, 1.60)	<0.001	1.62 (1.60, 1.63)	<0.001	3.34 (3.29, 3.40)	<0.001	3.22 (3.16, 3.27)	<0.001
Distant	6.60 (6.53, 6.67)	<0.001	6.99 (6.91, 7.08)	<0.001	17.98 (17.68, 18.28)	<0.001	16.98 (16.68, 17.29)	<0.001
*Tumour grade* ^‡^								
I	Reference		Reference		Reference		Reference	
II	1.15 (1.14, 1.17)	<0.001	1.07 (1.06, 1.09)	<0.001	1.40 (1.37,1.43)	<0.001	1.16 (1.14, 1.18)	<0.001
III	1.75 (1.72, 1.78)	<0.001	1.38 (1.35, 1.40)	<0.001	2.54 (2.49, 2.60)	<0.001	1.64 (1.60, 1.67)	<0.001
IV	1.86 (1.78, 1.93)	<0.001	1.54 (1.48, 1.60)	<0.001	2.65 (2.53, 2.78)	<0.001	1.82 (1.73, 1.91)	<0.001
*Chemotherapy*								
Yes	Reference		Reference		Reference		Reference	
No	0.83 (0.83, 0.84)	<0.001	1.13 (1.12, 1.15)	<0.001	1.85 (1.83, 1.87)	<0.001	1.06 (1.05, 1.07)	<0.001
*Surgery*								
Yes	Reference		Reference		Reference		Reference	
No	4.41 (4.43, 4.49)	<0.001	2.59 (2.54, 2.63)	<0.001	5.42 (5.32, 5.52)	<0.001	2.62 (2.56, 2.67)	<0.001
*Radiation therapy*								
Yes	Reference		Reference		Reference		Reference	
No	0.82 (0.80, 0.83)	<0.001	0.91 (0.90, 0.93)	<0.001	0.61 (0.59, 0.62)	<0.001	0.86 (0.84, 0.88)	<0.001

^‡^I means well differentiated; II means moderately differentiated; III means poorly differentiated; IV means undifferentiated. AP: Asian or Pacific Islander; AI/AN: American Indian/Alaska Native.

**Table 3:** Crude and adjusted hazard ratios for overall survival between right- and left-sided cancers by year, age, stage, and therapy.

Cohort	Crude HR (95% CI)	*P*	Adjusted HR^∗^ (95% CI)	*P*
*Year at diagnosis*				
All	0.86 (0.85, 0.87)	*<0.001*	0.92 (0.92, 0.93)	*<0.001*
1973–1982	0.88 (0.86, 0.90)	*<0.001*	0.96 (0.94, 0.98)	*<0.001*
1983–1992	0.91 (0.89,0.94)	*<0.001*	0.98 (0.97,1.00)	*0.059*
1993–2002	0.89 (0.87,0.91)	*<0.001*	0.95 (0.94,0.97)	*<0.001*
2003–2012	0.83 (0.81, 0.84)	*<0.001*	0.91 (0.89,0.92)	*<0.001*
*Age at diagnosis*				
All	0.86 (0.85, 0.87)	*<0.001*	0.92 (0.92, 0.93)	*<0.001*
<50 y	0.89 (0.86, 0.92)	*<0.001*	0.88 (0.85, 0.91)	*<0.001*
50–69 y	0.87 (0.86, 0.88)	*<0.001*	0.89 (0.88, 0.91)	*<0.001*
70 y	1.00 (0.99, 1.01)	*0.423*	1.02 (1.00, 1.03)	*0.006*
*SEER stage*				
All	0.86 (0.85, 0.87)	*<0.001*	0.92 (0.92, 0.93)	*<0.001*
Localized	0.81 (0.79, 0.82)	*<0.001*	0.99 (0.98,1.01)	*0.323*
Regional	0.90 (0.89,0.91)	*<0.001*	0.99 (0.98,1.00)	*0.156*
Distant	0.80 (0.79,0.82)	*<0.001*	0.81 (0.79,0.82)	*<0.001*
*Surgery*				
Yes	0.85 (0.85–0.86)	*<0.001*	0.92 (0.92, 0.93)	*<0.001*
None	0.81 (0.75–0.88)	*<0.001*	0.82 (0.79–0.85)	*<0.001*
*Radiation*				
Yes	0.54 (0.52–0.56)	*<0.001*	0.69 (0.66–0.72)	*<0.001*
None	0.86 (0.85–0.86)	*<0.001*	0.94 (0.93–0.94)	*<0.001*
*Chemotherapy*				
Yes	0.87 (0.86–0.88)	*<0.001*	0.83 (0.82–0.84)	*<0.001*
None/unknown	0.85 (0.84–0.85)	*<0.001*	0.96 (0.95–0.97)	*<0.001*

HR: hazard ratio; CI: confidence interval. ^∗^Adjusted for age, gender, race, year, insurance status, marital status, grade, surgery, radiation, and SEER stage. Right-sided cancer as conference.

## Data Availability

All data used to support the findings of this study are public.

## Abbreviations

SEER: Surveillance, Epidemiology, and End Results
HR: Hazard ratio
CI: Confidence interval
RCC: Right-sided colon cancer
LCC: Left-sided colon cancer
OS: Overall survival
CSS: Cancer-specific survival.
